# Multi-Target State and Extent Estimation for High Resolution Automotive Sensor Detections

**DOI:** 10.3390/s22218415

**Published:** 2022-11-02

**Authors:** Andinet Hunde

**Affiliations:** Department of Automotive Engineering, Clemson University, Clemson, SC 29634, USA; ahunde@clemson.edu

**Keywords:** multi-target tracking, extended object tracking, group tracking

## Abstract

This paper discusses the perception and tracking of individual as well as group targets as applied to multi-lane public traffic. Target tracking problem is formulated as a two hierarchical layer problem—on the first layer, a multi-target tracking problem based on multiple detections is distinguished in the measurement space, and on the second (top) layer, group target tracking with birth and death as well as merging and splitting of group target tracks as they evolve in a dynamic scene is represented. This configuration enhances the multi-target tracking performance in situations including but not limited to target initialization(birth), target occlusion, missed detections, unresolved measurement, target maneuver, etc. In addition, group tracking exposes complex individual target interactions to help in situation assessment which is challenging to capture otherwise.

## 1. Introduction

Advances in automotive sensing technology and the ever-increasing demand for environmental perception have stimulated researchers and practitioners alike in the development of tracking algorithms that address the accuracy and computational requirements of autonomous vehicles, while sophisticated tracking algorithms as demonstrated in airborne/ground target tracking applications underwent profound treatment following world war II, the presence of clutter, false alarms, missed detections as well as maneuvering targets pose challenges in tracking. Perception and tracking in the automotive industry are no different. In fact, owing to a significant leap in the development of automotive sensors, such sensors as RADAR and LIDAR can generate multiple detections/returns making measurement-to-track data associations even more complicated. The improved sensor resolution has revitalized research in the tracking of extended and group targets. In this paper, we focus on the perception and tracking of individual as well as group targets as applied to multi-lane public traffic. We formulate the tracking problem as a two hierarchical layer: at level 1, we distinguish multi-target tracking based on multiple detections represented in the measurement space. A situation assessment layer at level 2 tracks group targets with birth and death as well as merging and splitting functionalities as they evolve. This arrangement enhances the multi-target tracking performance in situations including but not limited to target initialization (birth), target occlusion, missed detections, unresolved measurement, target maneuver, etc. In addition, group targets expose complex individual target interactions to help in a situation assessment study which are otherwise challenging to capture.

In short, we monitor both the group and individual extended object (EO) tracks from sensors’ detections mounted on the moving ego vehicle. In addition to group behavior, which is directly observable from sensor detections, the behavior of individual EO is also equally important both in the short and long horizon control decisions. For instance, due to transient occlusions, detections from an EO might be missing over consecutive scan times. The group state that the target was known to be a part of can be used to complement missing detections, i.e., the group state essentially plays a role of a virtual measurement to update target state predictions of the EO. Furthermore, before an EO (or groups of EO) splits away from a group that is being tracked, the tendency for such an event (splitting, in this case) together with the time history of the trajectory before and following the event, can be used to make informed decisions about the evolving traffic. Some of these decisions could be critical such as when the splitting EO (a group of EO) ultimately joins the current or planned lane of the ego vehicle. In general, group tracking can improve individual EO tracking performance under missing detections, merged detections, and data association uncertainties.

Next, we shall highlight the objective of group tracking and comment on how it fits into the overall scheme of multi-target tracking. In short, the relevance of group tracking, especially under EO (multiple detections) scenarios is evident in four main areas:

- Data Association: The advent of high-resolution automotive sensors resulted in multiple detections which are distributed over the extent of a target vehicle. This in turn necessitated the use of algorithms capable of extended object tracking, which is a departure from conventional approaches to point target tracking that assumes at most one detection per object vehicle. The data association was simpler: the detection could be from a target or a nearby non-target or clutter. Under multiple detections, the sheer amount of data association that needs to be resolved especially under groups of extended objects poses a computational challenge. With group tracking, we can simplify the data association problem to mutually exclusive regions of clusters of detections which are treated as independent group detections. By way of contrast, the complexity of data association over a cluster of detections is less demanding than solving the same over all the measurement space.
- Merged Measurement: Even under EO considerations, cases of unresolved measurement (also called merged measurement) can be a challenging problem in multi-target tracking problems. A Merged measurement occurs when the sensor returns a single detection for multiple objects in the scene. This is mainly due to sensor imperfections and poor resolution for sufficiently distant detections. Generally, modeling and integrating merged measurements into the tracking scheme, improve tracking by preventing premature track terminations. However, this comes with an added computational complexity. The literature under merged measurement tracking cases is very rare, often ignored under the independent measurement assumption. The earliest work that models merged measurements and integrates it into the Joint Probabilistic Data Association (JPDA) algorithm is presented in [1]. In [2], a resolution model is developed and implemented within the Multiple Hypothesis Tracker (MHT) method. The standard sensor measurement is generalized to the case of merged measurement and then implemented into the generalised labelled multi-Bernoulli(GLMB) filter [3]. Instead of explicitly modeling the case of merged measurements, we intend to track the group targets for which merged measurements are reported until enough resolutions are obtained to separate them into independent tracks.
- Missing Detections: The rationale for using the group state as a virtual measurement to update the target track (with missing detections) which is known to be a member of a group is discussed above.
- Track Initialization: To start a new target track, we employ a one-point track initialization method. Furthermore, any detections which are not associated with a confirmed track are used to initiate tentative tracks. The target velocities and accelerations are assumed to be $0_{d,1}$, where d∈{2,3} is the dimension of the target track. Both state variables are estimated from filtering recursion which takes a few measurement scans to settle into a reasonable estimation. We also intend to improve the estimation by feeding the average velocities and accelerations of groups in the FoV of the ego vehicle to initialize the states. The suggestion to use group velocities and accelerations so as to initialize new target track states is mentioned in [4].

**Figure 1:**
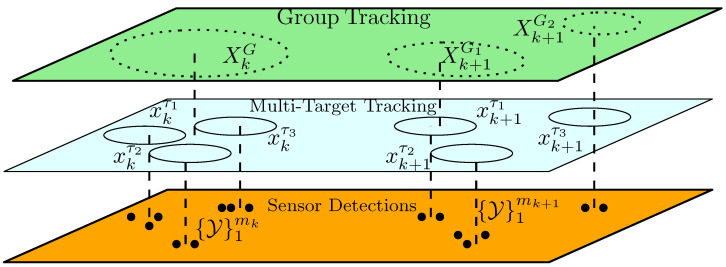
A double layer tracking scheme. The top layer tracks a set of target vehicles as a group, while the middle layer handles multi-target tracking as independent agents. Here, dots represent sensor detections, whereas solid and dotted ellipses respectively represent the extent of individual and group targets.

Before closing this section, we shall clarify the use of the terms “extended” and “groups” targets which are often interchangeably used in the literature without distinctions. From the viewpoint of sensor detections, an extended object tracking problem is closely related to group tracking, but there are multiple important differences. Groups have an internal degree of freedom that affects the shape of the group whereas the extent of EO is relatively fixed in most circumstances and for the most part of its motion. Moreover, groups possess unique behaviors of splitting and merging which, generally, are not characteristics of an EO. It also needs to be clarified that the objective here is not to model inter-group interactions, but rather to focus on the use of the group behavior in improving individual EO tracking performance. In that sense, we propose a hierarchical structure with feedback from the group to individual track layers arranged as shown in Figure 1. The hierarchical arrangement is first proposed in [5], but with a different objective and chiefly from the viewpoint of modeling abstractions.

## 2. Related Work

The self-organizing ability of vehicular traffic is the result of intelligent decisions by autonomous agents and/or human-driven vehicles. These decisions are influenced by the desire to avoid collision while navigating a structured multi-lane road network (repulsion) and by the intention to reach a common goal such as driving through an exit (cohesion) [6], while individual behavioral responses can be modeled and predicated when taken in isolation; the aggregate dynamics is remarkably complex and often unpredictable. To this end modeling approximations exist that range from a microscopic viewpoint captured by a system of ordinary differential equations to a kinetic theory description via mean-field limit and a macroscopic level via a suitable hydrodynamic approximation, for more details on this topic please see the discussions in [7]. In particular, the hydrodynamic model that presupposes the continuity assumption cannot be applied to traffic flow. This is due to the small number of participants even in the case of traffic jams to justify the analogy with particle flows in fluid dynamics. Similarly, the kinetic theory is criticized for not taking into account the fact that a vehicle is not a particle, but rather an intelligent entity linking a driver and mechanics and hence the driver’s reaction needs to be considered [8].

By far, a theoretically unified and a rigorous framework for group detection, tracking, and identification is presented by Mahler [5]. Using “finite-set statistics” (FISST), a theoretically optimal recursive Bayes filter for the multisensor-multigroup problem, which is cast as a three-level statistical model, is constructed. Since the resulting filtering equations are computationally daunting even for the simplest of expected tracking problems, the author proposes computationally tractable approximation strategies by generalizing the concept of a probability hypothesis density (PHD) filter.

In [4], a dynamical model and Bayesian filtering algorithm are presented for detection and tracking of group and individual targets. The mathematical model is based on discrete stochastic differential equations that imitate the behavioral properties of biological groups. Repulsive forces are introduced to model closely spaced targets and to prevent an unintended collision. The resulting distribution for the dynamical and observation model is seen to be complex and highly nonlinear. As a result of which, a Markov chain Monte Carlo (MCMC) approach is implemented to perform sequential inference. Although the proposed model seeks to capture target interactions, there are some limitations that could prohibit its use in tracking groups of extended objects. First, the observability of all of the individuals within the group is very questionable. Second, the high dimension of the joint target state that increases sN times as the number of targets (*N*) increases could be a challenge in real-time applications. Here, *s* is the dimension of the state variables. A group tracking scheme, similar in spirit to [4], that jointly estimates the group structure, as well as the group states based on evolving networks and Monte Carlo methods, is presented in [9]. The nodes in the graph correspond to targets within the group and connected components correspond to groups of targets. Further studies that incorporate group structure into the joint state estimation scheme include the works in [10,11]. Both [10,11] build on a presupposition that fixes the maximum number of groups anticipated in the tracking scene.

The work in [12] presents a performance comparison of three cluster tracking techniques. These are the independent Sampling Importance Resampling (SIR) PFs, an extended object PHD filter, and a Gaussian mixture Markov chain Monte Carlo (MCMC). It is shown that the MCMC approach exhibits the best tracking accuracy, essentially yielding the least number of false detections. Further, efficient SMC implementations, both from algorithmic and hardware implementation view points, are discussed to make SMC methods suitable for high-dimensional problems and real-time applications. In [13], a filtering algorithm based on a Markov chain Monte Carlo (MCMC) for tracking multiple clusters of coordinated objects is presented. A dynamic Gaussian mixture model is utilized for representing the time-varying clustering structure. This involves point process formulations of typical behavioral moves such as birth and death of clusters as well as merging and splitting.

The measurement model that extends the point-source model to multiple detections of EO based on Poisson target-measurement model was discussed in [14,15]. The Poisson process assumption with a spatially dependent rate parameter allowed several measurements to originate from the target and the extent to be seen as a spacial probability distribution. The most common approach in EO and group target tracking considers an augmented state that jointly estimates the position of group center and its extent via either random hyper-surface or the random matrix approach. For the detailed treatment of the two approaches, see the references [16,17,18,19,20,21,22,23]. In general, in the approach based on the random matrices, the extent is considered to be a random process and hence is normally assigned a corresponding prior (e.g., Wishart distribution [19,20,21]) and a transition kernel. In [24], in order to improve the estimation performance of interacting multiple model (IMM) tracking algorithm for group targets, two variable structure IMM algorithms are presented within the random matrices framework. A similar effort that uses the multiple model structure to improve the Gamma Gaussian inverse Wishart probability hypothesis density (GGIW-PHD) filter algorithm is also proposed in [25]. The multiple model structure is built into the estimation of kinematic state and extension state and is meant to improve the tracking performance during the maneuvering stage.

## 3. Objectives and Contributions

Our objective is to improve the tracking performance of individual extended objects during common public traffic events involving occlusions, track initiation, and merged measurements. To that end, we propose a hierarchical tracking structure where the lower layer deals with the multi-detection multi-target track estimation of individual extended objects while the upper layer executes group tracking to facilitate a feedback mechanism that provides group state to the lower tracking layer.

The specific objective at the upper layer is not to model inter-group interactions, but rather to emphasize the use of group behavior as a mechanism to enhance the performance of individual target tracks via feedback to the lower layer. This approach was first suggested in [26] and was identified to have the best potential for accurate tracking performance among the three methods compared in the article.

### Contributions

In order to address the large data association uncertainty in the presence of high-resolution detections, we use the linear multi-target (LM) Integrated Probabilistic Data Association (IPDA) approach for handling the data association problem. The joint kinematic and extent estimation is facilitated through the random matrix approach outlined in [21]. The work that combines the LMIPDA approach as applied to IPDA is not reported so far. In addition, the hierarchical scheme that combines the LMIDPA and random matrix as a joint extended target state at each of the two layers with the objective of improving the tracking performance is not presented elsewhere. The resulting method will be subject to challenging simulated scenarios and real radar detections obtained from experimental data to investigate its performance and utility. Elsewhere, the multi-detection Joint integrated track splitting (MD-JITS) filter is combined with the random matrix extent estimation technique in [27]. In [28], for the data association problem, a generalized probabilistic data association filter is applied. Although the data association uncertainties are handled by a closely related method, both [27,28] are confined to the discussion of individual extended target tracking problems.

## 4. Multiple Detection and Extended Object (Group) Tracking

For extended target tracking, the joint density of the kinematic state $x_{k}$ and the objection extension $X_{k}$ are iteratively computed. In Bayesian filtering recursion, the joint target density $p(x_{k},X_{k}|Z^{k})$ undergoes a prediction step followed by a measurement update. The prediction step is based on an assumed kinematic/dynamic evolution model that approximates the motion of the target.

(1) $$p\left(x_{k-1},X_{k-1}|Z^{k-1}\right)\to p\left(x_{k},X_{k}|Z^{k-1}\right)$$

which can be interpreted as a marginal density integrated as [19]:

(2) $$\begin{array}{c} p\left(x_{k},X_{k}|Z^{k-1}\right)=\int dx_{k-1}dX_{k-1}\times \\ p(x_{k},X_{k}|x_{k-1},X_{k-1},Z^{k-1})\times \\ p(x_{k-1},X_{k-1}|Z^{k-1}) \end{array}$$

The transition density $p(x_{k},X_{k}|x_{k-1},X_{k-1},Z^{k-1})$ can be written as a product of kinematic and object evolution sub-parts:

(3) $$\begin{array}{c} p(x_{k},X_{k}|x_{k-1},X_{k-1},Z^{k-1})=\\ p(x_{k}|X_{k},x_{k-1},X_{k-1},Z^{k-1})\times \\ p(X_{k}|x_{k-1},X_{k-1},Z^{k-1}) \end{array}$$

We make use of Markov-type assumptions for its kinematical part, i.e., $p(x_{k}|X_{k},x_{k-1}$, $X_{k-1},Z^{k-1})=p\left(x_{k}|X_{k},x_{k-1}\right)$ and assume that the object’s kinematical properties have no impact on the temporal evolution of the object extent and previous measurements if $X_{k-1}$ is given, i.e.:

(4) $$p(X_{k}|x_{k-1},X_{k-1},Z^{k-1}=p\left(X_{k}|X_{k-1}\right)$$

We thus have:

(5) $$p\left(x_{k},X_{k}|x_{k-1},X_{k-1},Z^{k-1}\right)=p\left(x_{k}|X_{k},x_{k-1}\right)p\left(X_{k}|X_{k-1}\right)$$

We now obtain the prediction formula:

(6) $$\begin{array}{c} p\left(x_{k},X_{k}|Z^{k-1}\right)=\int dx_{k-1}dX_{k-1}\\ p\left(x_{k},X_{k},x_{k-1}\right)p\left(X_{k}|X_{k-1}\right)\times \\ p\left(x_{k-1}|X_{k-1},Z^{k-1}\right)p\left(X_{k-1}|Z^{k-1}\right) \end{array}$$

Further discussion is much simplified if the temporal evolution of the object extension is assumed to have no effect on the prediction of the kinematic object properties. That is, we can make the assertion $p\left(x_{k-1}|X_{k-1},Z^{k-1}\right)\approx p\left(x_{k-1}|X_{k},Z^{k-1}\right)$ or, in simple terms, we intend to replace $X_{k-1}$ by $X_{k}$. We can write, from Bayes theorem, the joint predicted density as given in Equation (7).

(7) $$p\left(x_{k},X_{k}|Z^{k-1}\right)=p\left(x_{k}|X_{k},Z^{k-1}\right)p\left(X_{k}|Z^{k-1}\right)$$

The two densities can then be independently integrated out from Equations (8) and (9).

(8) $$\begin{array}{c} p\left(x_{k}|X_{k},Z^{k-1}\right)=\int p\left(x_{k}|X_{k},x_{k-1}\right)\times \\ p\left(x_{k-1}|X_{k},Z^{k-1}\right)dx_{k-1} \end{array}$$

(9) $$p\left(X_{k}|Z^{k-1}\right)=\int p\left(X_{k}|X_{k-1}\right)p\left(X_{k-1}|Z^{k-1}\right)dX_{k-1}$$

The update step incorporates new data ($Z_{k}$) and propagates the sensor prediction to time $t_{k}$ as follows.

(10) $$p\left(x_{k},X_{k}|Z^{k-1}\right)\to p\left(x_{k},X_{k}|Z^{k}\right)$$

Given the measurement likelihood defined as $p(Z_{k},m_{k}|x_{k},X_{k})$ and the predicted density of Equation (7), we can write the update step as shown in Equation (11).

(11) $$p\left(x_{k},X_{k}|Z^{k}\right)=\frac{p\left(Z_{k},m_{k}|x_{k},X_{k}\right)p\left(x_{k},X_{k}|Z^{k-1}\right)}{\int p\left(Z_{k},m_{k}|x_{k},X_{k}\right)p\left(x_{k},X_{k}|Z^{k-1}\right)dx_{k}dX_{k}}$$

### 4.1. Bayesian Extended Object Tracking

We assume that the sensor detections include a set of position measurements in two dimensions (x−y plane). However, the kinematic state variable corresponds to velocity and acceleration in addition to the position states.

#### 4.1.1. Measurement Model

At each measurement scan *k*, a random number of measurements $n_{k}$ labeled as $y_{k}^{i}$ where $i\in 1,2,...,n_{k}$ are collected from the sensor [21].

(12) $$y_{k}^{i}=Hx_{k}+w_{k}^{i}$$

Let $Y_{k}={\left\{y_{k}^{i}\right\}}_{i=1}^{n_{k}}$ and $Y_{k}:={\left\{Y_{t},n_{t}\right\}}_{t=0}^{k}$. The noise $w_{k}^{i}$ takes the form of a Gaussian density with zero mean and variance $X_{k}$. For the measurement set $Y_{k}$, the likelihood is computed as in Equation (13).

(13) $$p\left(Y_{k}|n_{k},x_{k},X_{k}\right)=\prod_{i=1}^{n_{k}}N\left(y_{k}^{i};Hx_{k},X_{k}\right)$$

For the set of measurements $Y_{k}$, define the mean and the measurement spread as in Equations (14) and (15).

(14) $${\bar{y}}_{k}=\frac{1}{n_{k}}\sum_{i=1}^{n_{k}}y_{k}^{i}$$

(15) $${\bar{Y}}_{k}=\sum_{i=1}^{n_{k}}\left\{y_{k}^{i}-{\bar{y}}_{k}\right\}{\left\{y_{k}^{i}-{\bar{y}}_{k}\right\}}^{T}$$

Equation (13), can be written as:

(16) $$\begin{array}{c} p\left(Y_{k}|n_{k},x_{k},X_{k}\right)\propto N\left({\bar{y}}_{k};Hx_{k},X_{k}/n_{k}\right)\times \\ W\left({\bar{Y}}_{k};n_{k}-1,X_{k}\right) \end{array}$$

where

(17) $$W\left(X;m,C\right)=\frac{{\left|X\right|}^{\frac{m-d-1}{2}}}{2^{\frac{md}{2}}\Gamma_{d}\left(\frac{m}{2}\right){\left|C\right|}^{\frac{m}{2}}}\exp\left(tr\left[-\frac{1}{2}XC^{-1}\right]\right)$$

with m≥d, W(X;m,C) is the Wishart density of a d-dimentional SPD random matrix *X* with an expected SPD matrix mC.

#### 4.1.2. Tracking Algorithm

Applying the Chapman-Kolmogorov theorem and the concept of conjugate priors, a recursive joint state estimation scheme is derived in [19]. The joint probability density is factored as follows.

(18) $$p\left(x_{k},X_{k}|Y^{k}\right)=p\left(x_{k}|X_{k},Y^{k}\right)p\left(X_{k}|Y^{k}\right)$$

Under the assumed probability density functions, this further results in

(19) $$p\left(x_{k},X_{k}|Y^{k}\right)=N\left(x_{k};x_{k/k},P_{k/k}⊕X_{k}\right)\mathrm{IW}\left(X_{k};\nu_{k/k},X_{k/k}\right)$$

where ⊕ represents the Kronecker product. The inverse Wishart density is parameterized as follows:

(20) $$W\left(X;m,C\right)=\frac{{\left|C\right|}^{\frac{m}{2}}}{2^{\frac{md}{2}}\Gamma_{d}\left(\frac{m}{2}\right){\left|X\right|}^{\frac{m+d+1}{2}}}\exp\left(tr\left[-\frac{1}{2}CX^{-1}\right]\right)$$

The authors in [21], proposed the measurement likelihood (13) to take the form of a Gaussian density as shown in Equation (21).

(21) $$p\left(Y_{k}|n_{k},x_{k},X_{k}\right)=\prod_{i=1}^{n_{k}}N\left(y_{k}^{i};Hx_{k},zX_{k}+R\right)$$

where, the overall covariance matrix is composed of the sensor error covariance matrix *R* and an additional term that includes the spread contribution of the object extension scaled by a factor of *z*. Further, from Equation (18), we approximate that:

(22) $$p\left(x_{k}|X_{k},Y_{k}\right)\approx p\left(x_{k}|Y_{k}\right)\approx N\left(x_{k};Hx_{k},zX_{k}+R\right)$$

where

(23) $$x_{k|k}=x_{k|k-1}+K_{k|k-1}\left({\bar{y}}_{k}-Hx_{k|k-1}\right)$$

(24) $$P_{k|k}=P_{k|k-1}-K_{k|k-1}S_{k|k-1}K_{k|k-1}^{T}$$

where

(25) $$S_{k|k-1}=HP_{k|k-1}H^{T}+\frac{Y_{k|k-1}}{n_{k}}$$

(26) $$K_{k|k-1}=P_{k|k-1}S_{k|k-1}^{-1}$$

(27) $$Y_{k|k-1}=zX_{k|k-1}+R$$

The extension update $p(X_{k}|Y_{k})$ is approximated as

(28) $$p\left(X_{k}|Y_{k}\right)\approx \mathrm{IW}\left(X_{k};v_{k/k},\alpha_{k|k}X_{k|k}\right)$$

where

(29) $$X_{k|k}=\frac{1}{\alpha_{k|k}}\left(\alpha_{k|k-1}X_{k|k-1}+{\hat{N}}_{k|k-1}+{\hat{Y}}_{k|k-1}\right)$$

(30) $$\alpha_{k|k}=\alpha_{k|k-1}+n_{k}$$

The authors in [21] assume independence between the kinematics and extent estimates. In addition, if the dynamic models for the kinematics and extent prediction are independent, we can use standard Kalman filter prediction equations as follows.

(31) $$x_{x|k-1}=Fx_{k-1|k-1}$$

(32) $$P_{x|k-1}=FP_{k-1|k-1}F^{T}+Q$$

(33) $$X_{x|k-1}=X_{k-1|k-1}$$

(34) $$\alpha_{k|k-1}=2+\exp\left(\frac{-T}{\tau }\right)\left(\alpha_{k-1|k-1}-2\right)$$

### 4.2. Group Tracking Algorithm

**Figure 2:**
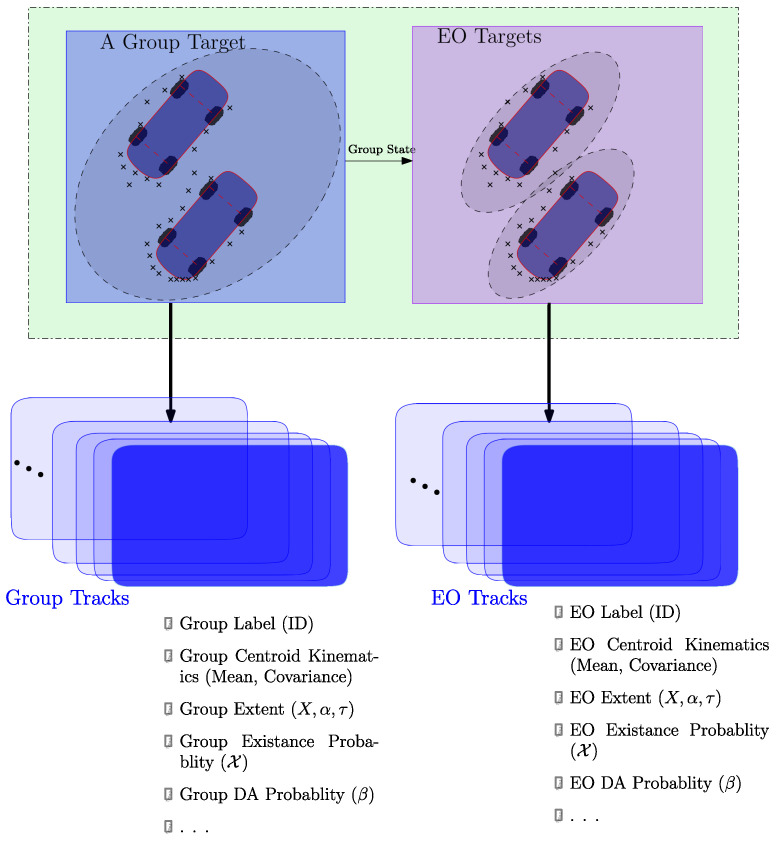
Groups of targets like individual EO target tracks go through birth/death process, i.e., can be initialized/terminated and maintained when updates are available.

In the hierarchical tracking scheme, the group tracks and the EO tracks share a similar structure in the manner they handle tracks. This is illustrated in Figure 2.

To improve the tracking performance of individual extended objects a hierarchical tracking structure is shown in Figure 2. The lower layer handles a multi-detection multi-target track estimation of individual extended objects and the upper layer executes group tracking to facilitate a feedback mechanism that provides group state to the lower tracking layer. At both layers, track management schemes handle track initiation, confirmation, and termination procedures. Tentative tracks are initialized on measurements not validated by any of the tracked targets. Furthermore, the prevalent assumption about track initiation is that our knowledge of the prior information about the tentative target velocity and acceleration vectors are limited to the maximum speed $v_{max}$ and the maximum acceleration $a_{max}$ [29,30]. In this approach, the initial state estimate and covariance matrix formulations follow a similar structure as presented in our previous works [31,32,33]. Here, the presence of other groups in the FoV of the ego vehicle can be used to improve our prior information on the velocity and acceleration of a tentative track.

**Figure 3:**
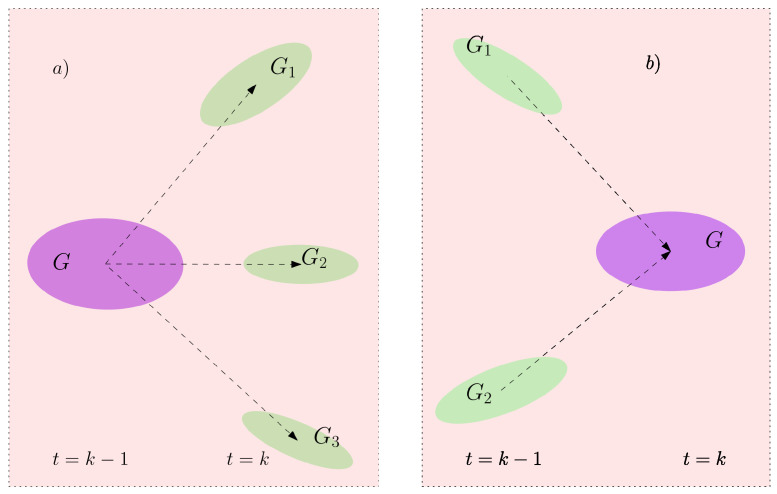
Unlike individual EO target tracks, group tracks (colored in purple) (**a**) can be split into smaller subgroups (colored in green) or (**b**) merge to form an even larger group.

In addition to the track initiation, maintenance and termination attributes that a group track shares with individual EO tracks, track splitting and merging events that are specific to group tracks need to be handled too, see Figure 3. Conceptually, the merging process terminates either of the sub-groups while initiating a new group track with a unique identity. The same logic applies to the splitting event. The parent group might survive into one of the splitting sub-groups or new tracks might be initiated for all the split-up sub-groups. Both, merging and splitting events are handled by the tracking algorithm which is based on LMIPDA.

## 5. Results and Discussions

In this section, we discuss and evaluate the performance of multi-detection algorithms on simulated and experimental target tracking scenarios. Since EO tracking considers the simultaneous estimation of the kinematic state and the shape parameters of a moving object, a performance metric that can measure both is required. For EO tracking, a distance measure should incorporate geometric shape [34,35]. We evaluate the location and extent errors simultaneously with a single score by means of the Gaussian Wasserstein distance as follows:

(35) $$\begin{array}{c} d\left(\mu_{1},\Sigma_{1},\mu_{2},\Sigma_{2}\right)=\left|\right|\mu_{1}-\mu_{2}\left|\right|+\\ tr\left\{\Sigma_{1}+\Sigma_{2}-2\sqrt{\sqrt{\Sigma_{1}}\Sigma_{2}\sqrt{\Sigma_{1}}}\right\} \end{array}$$

where $\mu_{1,2},\Sigma_{1,2}$ are, respectively, the mean vector and the co-variance matrix of Gaussian distributions and $\mu_{1},\mu_{2}\in R^{2}$, $\Sigma_{1},\Sigma_{1}\in R^{2\times 2}$.

### 5.1. Evaluations on Simulated Data

**Figure 4:**
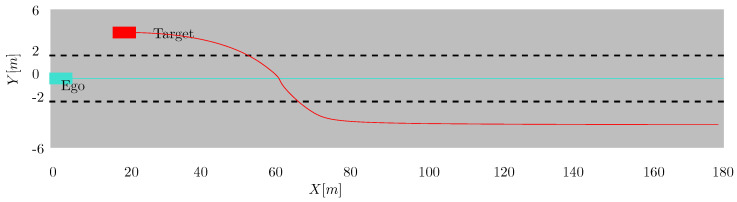
A maneuvering vehicle executes a lane change in front of the ego vehicle which is equipped with a high resolution sensor.

A single target in front of the ego vehicle executes a lane change manoeuvre from the left to the right lane. Both the target and the ego vehicle are traveling at a constant speed of 20 m/s. Initially, the target vehicle is 20 m ahead of the ego vehicle in the middle of the left lane (y = 4 m). The approximate trajectory that both vehicles traverse is shown in Figure 4. The measurement is assumed to have a Poisson distribution with a known rate.

#### 5.1.1. The Case of Low Measurement Density, λ=5

**Figure 5:**
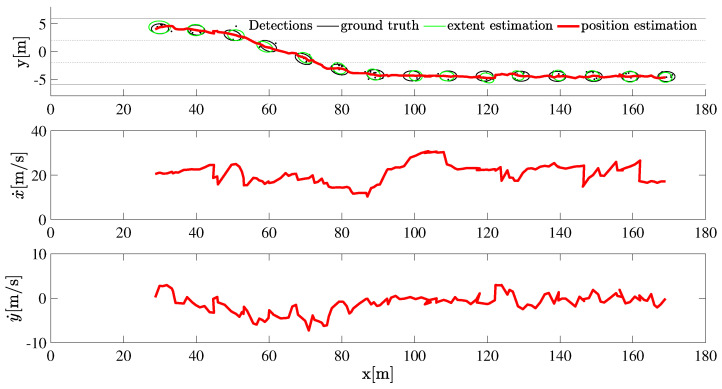
The tracking result of a maneuvering target with a measurement distribution assumed to be Poisson with parameter λ=5.

**Figure 6:**
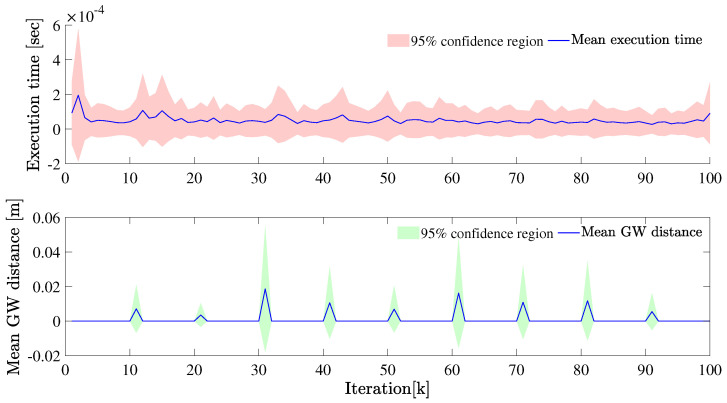
The mean GW distance and execution time at each iteration is plotted for λ=5.

First, we fix Poisson distribution with parameter λ=5. The tracking result is given in Figure 5 and Figure 6. Compared to the ground truth, the extent estimation as well as the filtered velocities show more deviation compared to the case when λ is higher.

#### 5.1.2. The Case of High Measurement Density, λ=50

**Figure 7:**
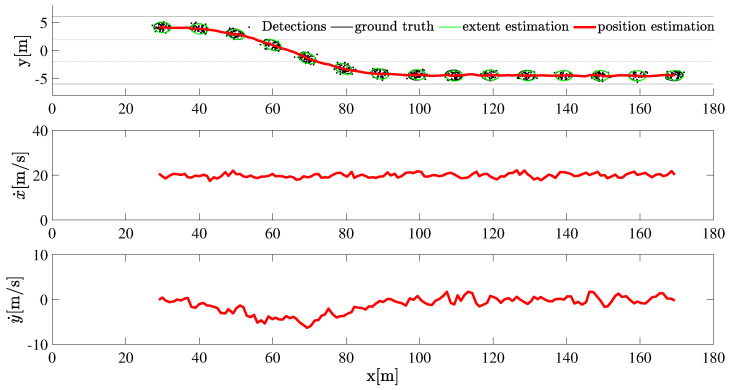
The tracking result of a maneuvering target with a measurement distribution assumed to be Poisson with parameter λ=50.

**Figure 8:**
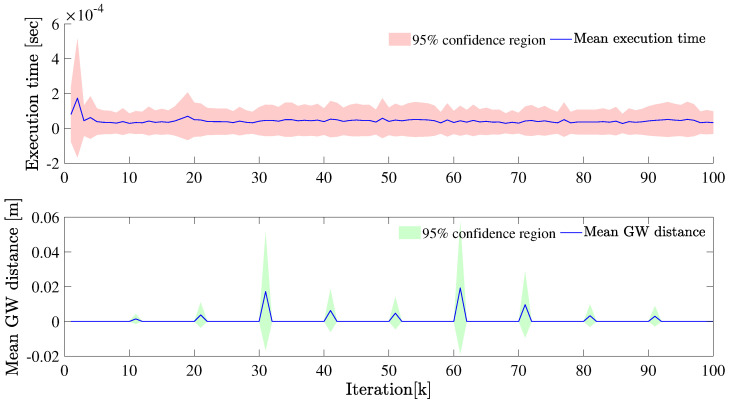
The mean GW distance and execution time at each iteration is plotted, λ=50.

Next, we fix the Poisson rate at λ=50. As seen in Figure 7 and Figure 8, the extent estimation is better than the case with λ=5. Because of the coupling of the kinematic state and extent, the filtered velocities are seen to be reasonably close to the ground truth values and show less deviation compared to the case when the λ=5.

#### 5.1.3. The Case of Missing Detections, without Group Information

**Figure 9:**
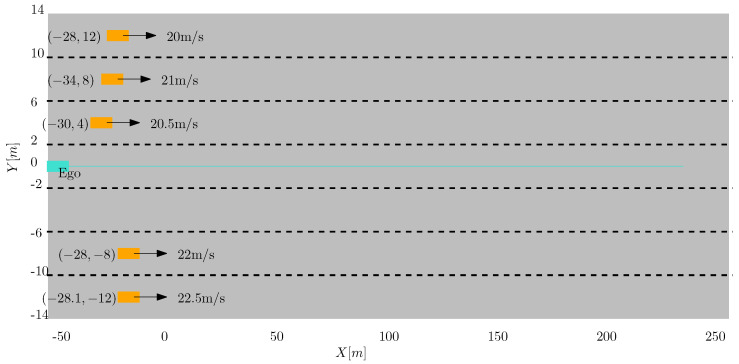
Scenario to demonstrate the use of group state information in the presence of occlusions.The target vehicles are colored orange, whereas the ego vehicle is shown in Turquoise.

**Figure 10:**
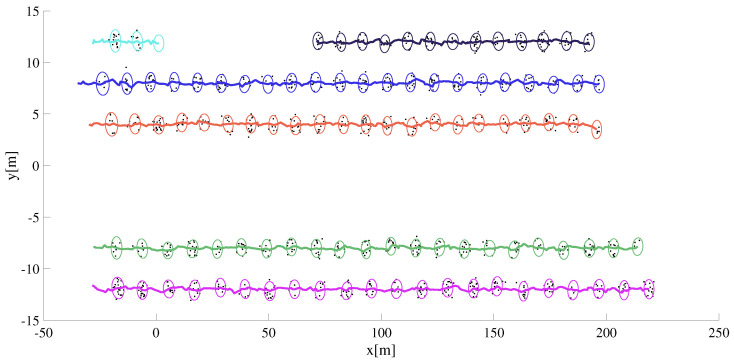
One of the EO has missing detections for a duration of 70 measurement scans. Its track is terminated and a new track is initiated when the target detections are available. Each target track is assigned to a unique color similar to the manner its track ID is assigned.

Figure 9 shows, a scenario where five extended objects are depicted deriving in close proximity and thus creating two groups of vehicles, three of them to the left and the remaining two to right of the ego vehicle. The initial configuration in terms of the relative position with respect to the ego vehicle and the absolute velocities of the EO is also given. We also simulate a scenario where due to an assumed occlusion, the detections from the leftmost EO are missing for 70 consecutive scans, i.e., from t=[1.55] seconds into the simulation. Without feedback from group tracking module, the tracks of the EO whose detections are missing is terminated. The track ID and only its attributes will be deleted as shown in Figure 10. When the occlusion is over and detections are available from the target, a new track is initiated. Even though the target vehicle is the same, two tracks are initiated with separate IDs because of the occlusion.

#### 5.1.4. The Case of Missing Detections, with Group Information

For the same scenario given in Figure 9, feedback from the group track layer of the proposed hierarchical scheme is made available to the EO multi-target tracking layer to illustrate its effectiveness. First, the group consisting of the left three extended objects is tracked for a while until the occlusion deemed the detections from the leftmost EO is unavailable. Since, the EO is known to be a member of this group, the group state is translated into a virtual measurement via the feedback path to update the target track. Here, for the virtual measurement we use:

(36) $$y_{k}^{\tau }=H_{pos}x_{k}^{\tau }+H_{vel}x_{k}^{G}dt$$

where, $H_{pos}$ and $H_{vel}$ select the position and velocity entries of the state variable, respectively.

(37) $$H_{pos}=\left[\begin{array}{cccccc} 1 & 0 & 0 & 0 & 0 & 0\\ 0 & 0 & 0 & 1 & 0 & 0 \end{array}\right]$$

(38) $$H_{vel}=\left[\begin{array}{cccccc} 0 & 1 & 0 & 0 & 0 & 0\\ 0 & 0 & 0 & 0 & 1 & 0 \end{array}\right]$$

**Figure 11:**
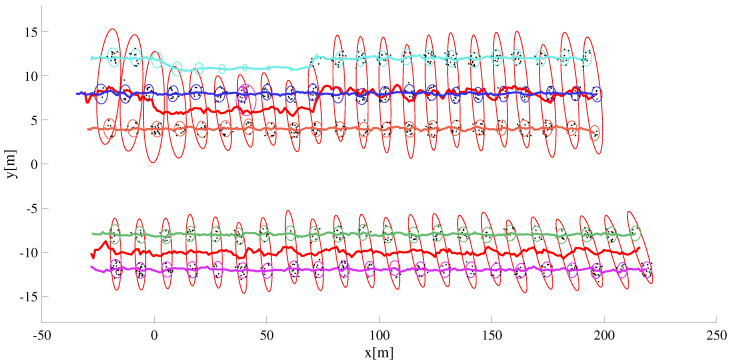
One of the EO has missing detections for a duration of 70 measurement scans. The group state information the EO is known to be a member of is used to update its track.

As shown in Figure 11, the group information is used to maintain the target track whose detections were missing for several measurement scans.In the figure, group extents and group position estimated are shown in red whereas each target track is assigned to a unique color similar to the manner its track ID is assigned.

### 5.2. Evaluations on Experimental Data

The mmwave radar sensor used in the both single EO and group EO target experiments has the specifications as shown in Table 1.

#### 5.2.1. Single Target Tracking

**Figure 12:**
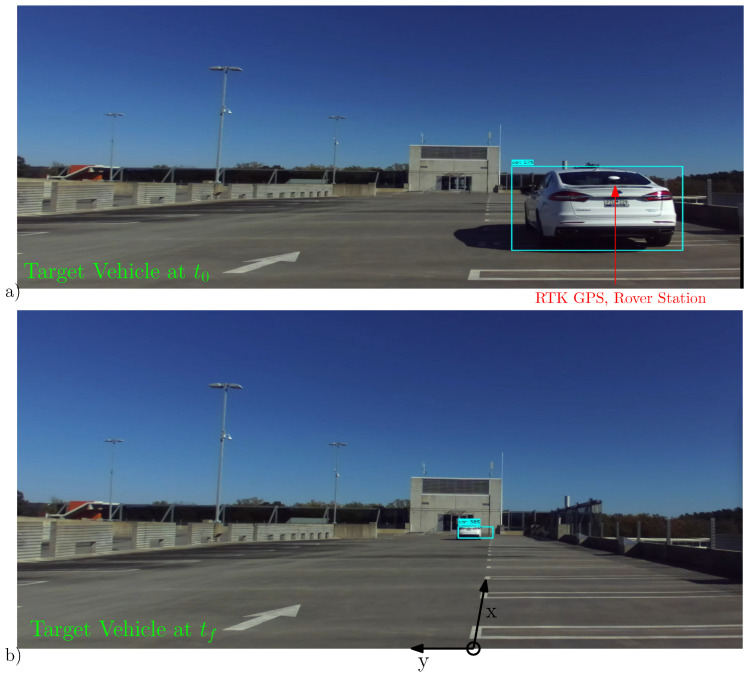
Pictured here is the experimental setup for a single target tracking scenario. (**a**) The target vehicle is shown at its starting pose at $t_{0}$. The rover station of the RTK GPS is also shown positioned at approximately the center of the rear axle. (**b**) The target vehicle is shown positioned at $t=t_{f}$. The positive x-y coordinate axes of the radar are also depicted.

The positive directions of the x-y coordinate axes of the radar sensor are established as shown in the experimental set up of Figure 12. On the other hand, the coordinate axes of the Real-time Kinematic (RTK) GPS is given in East-North-Up system, while the base station unit of the RTK GPS is fixed close to the radar coordinate frame, a suitable transformation is needed to align the East-North-Up readings to the local coordinate axes.

The rover unit of the RTK GPS is mounted on the target vehicle; logging positional data at a rate of 10 Hz, will serves as the ground truth. Radar point cloud detections are sampled and recorded on a tracking computer at a rate of 20 Hz. Ultimately, the GPS data is transformed to the local sensor frame coordinates and the GPS and radar timestamps are synchronized to use the RTK GPS location data to validate the performance of the tracking algorithms.

**Figure 13:**
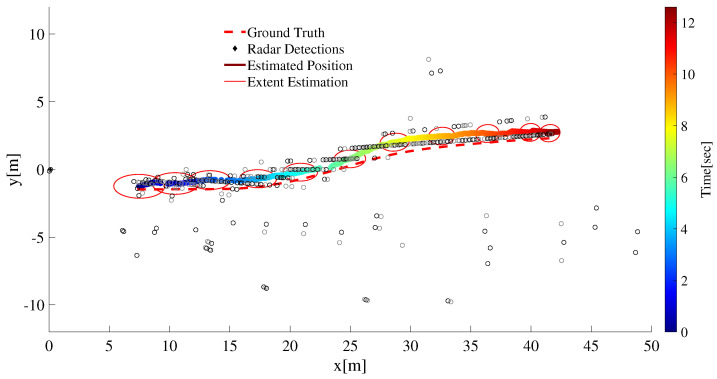
An extended target tracking result. The extent estimation is better with a high enough maneuver time constant θ=100Δt to compensate for less target detections at the far end of the track.

As shown from the pose of the target at $t_{0}$ and $t_{f}$ in Figure 13, the target vehicle executes a lane-change maneuver as it moves away from the ego vehicle. The kinematic and state estimation result is shown in Figure 13.

**Figure 14:**
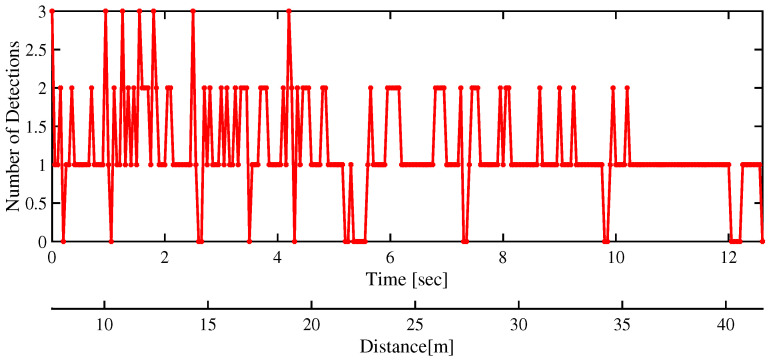
The number of radar target detections confirmed for the target are shown plotted as a function of its distance from the ego vehicle. As the distance increases the number of detections is seen to correspondingly decrease.

In Figure 14, the number of radar returns from the target is seen to decrease as the target moves away from the ego vehicle. For the extent estimation, a large enough value for the maneuvering time constant is chosen. This is particularly important considering the fact that the extent of the target remains approximately fixed. In addition, the extent estimation is improved because of a larger value of θ=100Δt chosen to compensate for less number of target detections at the far end of the track. It is also noted that, occasionally detections are missing and thus the track management part of the tracking algorithm is expected to maintain the target track.

**Figure 15:**
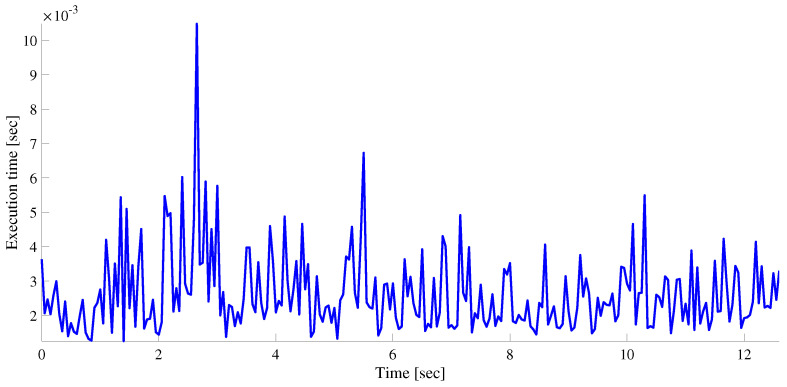
Execution time required to complete a single iteration for the case of a single ET.

In Figure 15, the time taken to execute a single iteration is plotted against time. Generally, the execution time depends on a number of factors, but most importantly on the number of radar detections and the number of targets (including false tracks which are based on ghost targets). We note that the mean and worst execution time per iteration are, respectively, 2.5 and 10.4 ms.

#### 5.2.2. Multi Target Tracking

Next, group target tracking is demonstrated in a multi-target scenario that involves two target vehicles. The availability of resources limit the number of targets to two, we expect the result to extend easily to scenarios involving more than two target vehicles. The reason being, the complex data association step that is computationally prohibitive as the number of target vehicles increases, is simplified in LMIPDA algorithm by its very design.

**Figure 16:**
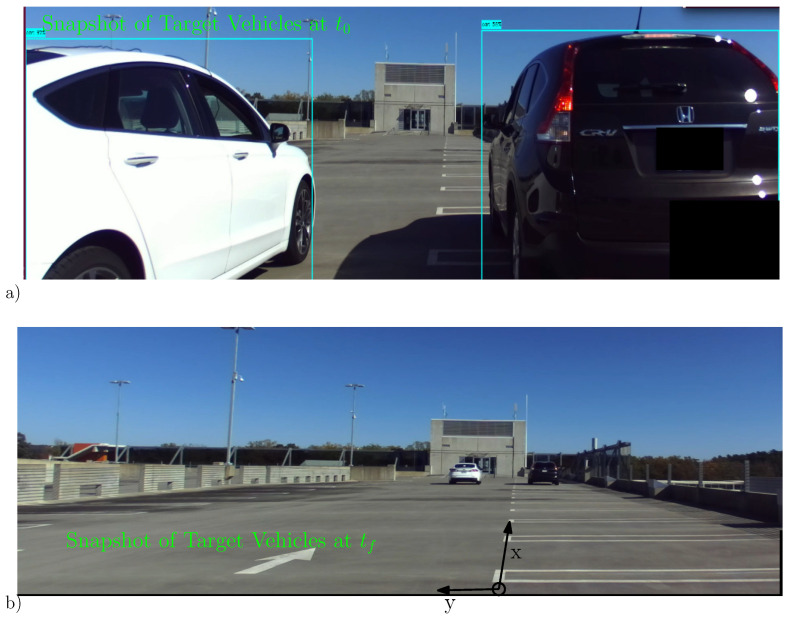
Experimental setup for the case of multiple ET target tracking scenario. (**a**) The target vehicles ($ET_{1}$(Sedan Car), $ET_{2}$(SUV Car)) are pictured at the starting pose $t=t_{0}$. The rover station of the RTK GPS is placed at the center of the rear axle of $ET_{1}$(Sedan Car). (**b**) The time-snapshot of both target vehicles is shown at $t=t_{f}$. The positive x-y coordinate axes of the radar sensor are also depicted.

**Figure 17:**
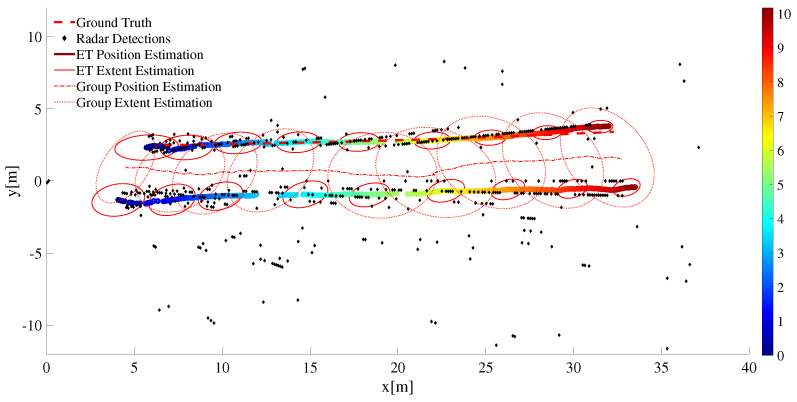
Tracking result of two extended targets. Both the position and extent estimation result for single ET and group target is shown.

In Figure 16, one of the target vehicles is equipped with RTK GPS to record the ground truth data, the other vehicle is merely there to facilitate the group target discussion (and to complicate the data association uncertainty). As seen from the time-snapshots at $t=t_{0}$ and $t=t_{f}$ of Figure 16, the two vehicles are driven in close proximity and in parallel as both drive away from the sensor station. The lateral inter-vehicle distance and the relative speed is intentionally kept reasonably close to simulate group target dynamics. The extent estimation for the individual ET as well as the group target is presented in Figure 17. In addition, the kinematic state estimate (for the position) is shown for both group and ET cases. Similar to the single ET case above, the maneuver time constant θ=100Δt is kept large enough to counter the extent estimation with fewer target detections.

The MD-LMIPDA tracker is able to resolve measurement-to-target data association without the need to compute the data association jointly for all the measurements and targets simultaneously. In addition, it is shown in Figure 17 that even under non-uniform and sparse target detections, extent estimations for both individual ET as well as group targets is possible. It is seen from the tracking discussions both under single target and multi targets that the algorithm can be used to successfully generate extended and group multi-target state estimates in public traffic scenarios.

**Figure 18:**
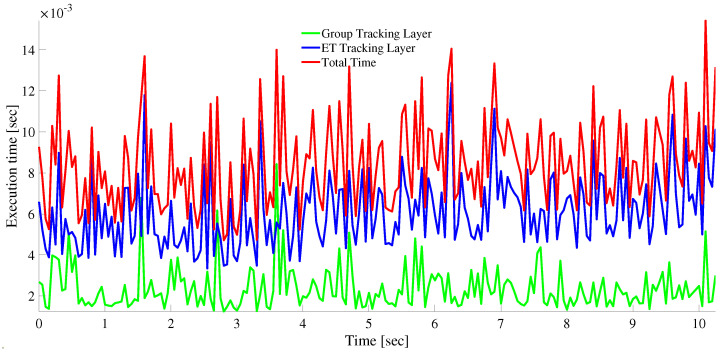
The execution time for per iteration for both group as well as ET tracking.

The applicability of the tracking algorithm under real-time requirement is further justified in the results plotted under Figure 18. As shown in the figure, the mean and maximum total execution time per iteration are, respectively, 8.5 and 19.8 ms. The total time is computed by simply adding the execution time for both group and ET tracking at a given radar scan time. In this work, all simulations are done on a laptop with the specifications: Intel Core i7 2.90 GHZ processor, installed Memory (RAM) of 16.0 GB and Windows 10 64-bit Operating system.

**Figure 19:**
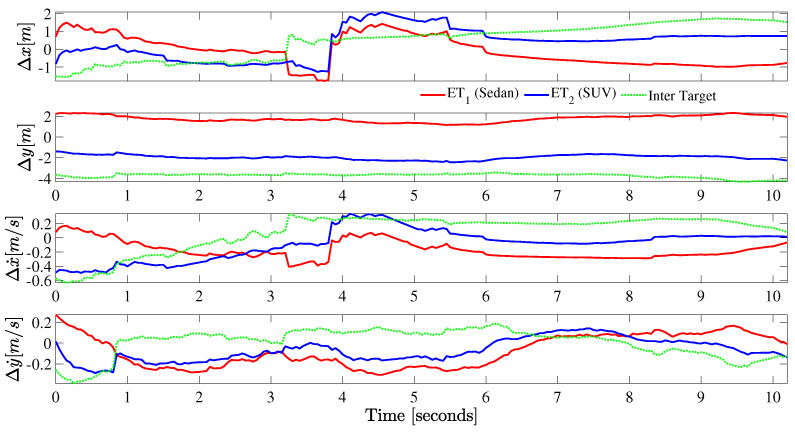
Relative distance and speed between the group and individual extended targets is plotted against time. The plots $ET_{1}$ and $ET_{2}$, measure the distance of individual target states from the group target. Whereas, the inter-target plots show the distance and speed differences between the two extended targets.

The notion of group objects is interpreted under stricter constraints of inter-target distances, relatives speeds and orientations. Targets in formation are required to have “similar” velocities and tight inter-object distances [4] to maximize the chances of being in a group. In Figure 19, the relative distance and speed between the group target and constituent extended targets is plotted against time. In addition, the inter-target distance and speed differences are shown on the same figure. Under the simulated scenario, a relative speed of $-0.4\leq \Delta \dot{y}\leq 0.2,-0.6\leq \Delta \dot{x}\leq 0.3$ and a relative distance of |Δy|≤4,|Δx|≤2 is observed.

## 6. Conclusions

The application of high-resolution automotive radar to public traffic presents a multi-detection tracking problem. In earlier work [36], we proposed a hybrid tracking scheme that exploits the measurement partitioning approach to address the tracking problem under multi-sensor multi-target scenarios. In the present paper, we proposed and demonstrated the use of group tracks to complement and improve individual extended object tracks under circumstances of missing detections, occlusions, target initialization and merged measurements. We used the Gaussian Wasserstein distance that incorporates both the shape and kinematic state in a single metric to evaluate the estimation performance of the tracking algorithm for various simulated examples.

The evaluation of the tracking algorithm for real radar detections is conducted for a single ET and promising results are obtained for real-time application. In particular, we noted the mean and maximum execution time per iteration in the order of 2.5 and 10.4 ms, respectively. A further experiment exploiting two extended targets was setup to emulate a dynamic group target. Radar detections from both target vehicles as well as RTK GPS data on one of the target vehicles is collected from two vehicles driving closely with each other and infront of the ego vehicle. The tracking problem is formulated as a two hierarchical layer: at the bottom layer, extended multi-target tracking algorithm is presented; at the top layer a group target tracking algorithm captures group evolution including merging and splitting of groups targets. The observed mean and maximum execution time per iteration are, respectively, 8.5 and 19.8 ms, respectively, justifying the use of the proposed tracking scheme for real-time applications.

More reliable extent estimation for both individual ET and group targets could be obtained under more dense sensor detections that also preferably take a uniform distribution across the extent of the ET and/or the group target. In this study, we employed an assumption on the extent evolution that tends to constrain the extent to vary only gradually. Practical traffic scenarios support this assumption: owing to the presence of structured lanes, tight traffic regulations and the inherent desire to avoid collisions, the extent varies rather slowly. This assumption also favors radar detections which tend to resemble a line segment or an “L-shape” even for high resolution options. Adding miss-detections and the possibility of relatively larger ego-to-target distances that reduce target detections, the extent estimation under the above assumption is clearly justified. However, for highly maneuvering targets and if the sole objective is to get reliable extent estimation, other sensors such as LiDAR and camera could be explored.

## Figures and Tables

**Table 1:** Radar parameters. This is the radar parameter and its setting as used for single EO and group EO experiments.

	Parameter	Value
1	Frequency Band	77–81 GHz
2	Azimuth Resolution	15 deg
3	Range Resolution	0.977 m
4	Max. Unambiguous Range	50 m
5	Max. Radial Velocity	18.55 m/s
6	Radial Velocity Resolution	0.29 m/s
7	Azimuth Resolution	15 deg

## Data Availability

The Data used in this work can be made available upon an email request to the corresponding author.
